# Biofluid-based predictors of post-concussion symptoms: a narrative review of mild traumatic brain injury biomarkers

**DOI:** 10.1093/braincomms/fcaf501

**Published:** 2025-12-18

**Authors:** Hannah S Lyons, Jessica C Hubbard, Chloe N Thomas, James A Roberts, Caroline W Mugo, Gabriel Bellamy Plaice, Olivia Grech, Sophie Prosser, Asha Strom, Samuel J E Lucas, Laura E Downie, Jessica M Gill, James L Mitchell, Alexandra J Sinclair, Lisa J Hill, UK mTBI-Predict Consortium, Adam Hampshire, Agata Czarnecka, Ahmed Fouad Abdel-Hay, Aimee R Smith, Alex Bryant, Alexandra J Sinclair, Ali Mazaheri, Alice J Sitch, Aliyah Mannan, Altus Chan, Andreas Yiangou, Andrew P Bagshaw, Andrew Palmer, Angus M Hunter, Animesh Ghose, Asha Strom, Caroline W Mugo, Carl R Krynicki, Caroline Witton, Cherie Nicholls, Chloe N Thomas, Claire H Brown, Clare Anderson, Dan Ford, Danny Smullen, David J Smith, David Jimenez-Grande, Davinia Fernandez-Espejo, Eleanor G Rowan-MacIndoe, Emma C Lardner, Hamid Dehghani, Hannah Fisher, Hannah S Lyons, Hyojin Park, Ian Varley, Jacob H Tennant, James L Mitchell, Jan Novak, Jennie Gavin, Jessica C Hubbard, John T Read, Jonthan J Deeks, Julita Sulkowska, Karen J Mullinger, Karen Tester, Katherine L Cox, Katie Morris, Linda Martina Coughlan, Lisa J Hill, Maria Balaet, Mark Thaller, Matt Hill, Mia Mann, Nasreen Akhtar, Ned J Jenkinson, Neil Winkles, Pete J Hellyer, Raymond F Reynolds, Richard J Blanch, Ryan S Ottridge, Sabrina Qureshi, Samuel J E Lucas, Sarah Berhane, Syama Mohan, Thomas Meredith, Tom Inns, Yousef F Hyder

**Affiliations:** Translational Brain Science, Department of Metabolism and Systems Science, College of Medicine and Health, University of Birmingham, Birmingham B15 2TT, UK; Department of Neurology, Queen Elizabeth Hospital, University Hospitals Birmingham NHS Foundation Trust, Birmingham B15 2GW, UK; Department of Biomedical Sciences, School of Infection, Inflammation and Immunology, College of Medicine and Health, University of Birmingham, Birmingham B15 2TT, UK; Department of Biomedical Sciences, School of Infection, Inflammation and Immunology, College of Medicine and Health, University of Birmingham, Birmingham B15 2TT, UK; Department of Biomedical Sciences, School of Infection, Inflammation and Immunology, College of Medicine and Health, University of Birmingham, Birmingham B15 2TT, UK; Translational Brain Science, Department of Metabolism and Systems Science, College of Medicine and Health, University of Birmingham, Birmingham B15 2TT, UK; Department of Biomedical Sciences, School of Infection, Inflammation and Immunology, College of Medicine and Health, University of Birmingham, Birmingham B15 2TT, UK; Department of Biomedical Sciences, School of Infection, Inflammation and Immunology, College of Medicine and Health, University of Birmingham, Birmingham B15 2TT, UK; Translational Brain Science, Department of Metabolism and Systems Science, College of Medicine and Health, University of Birmingham, Birmingham B15 2TT, UK; School of Sport, Exercise and Rehabilitation Sciences, University of Birmingham, Birmingham B15 2TT, UK; School of Sport, Exercise and Rehabilitation Sciences, University of Birmingham, Birmingham B15 2TT, UK; School of Sport, Exercise and Rehabilitation Sciences, University of Birmingham, Birmingham B15 2TT, UK; Department of Optometry and Vision Sciences, The University of Melbourne, Melbourne, Victoria 3010, Australia; School of Nursing, Johns Hopkins University, Baltimore, MD 21205, USA; Department of Neurology, Queen Elizabeth Hospital, University Hospitals Birmingham NHS Foundation Trust, Birmingham B15 2GW, UK; Department of Biomedical Sciences, School of Infection, Inflammation and Immunology, College of Medicine and Health, University of Birmingham, Birmingham B15 2TT, UK; Academic Department of Military Rehabilitation, Defence Medical Rehabilitation Centre, Loughborough LE12 5QD, UK; National Institutes of Health and Care Research Birmingham Biomedical Research Centre, University Hospitals Birmingham, Birmingham B12 2TH, UK; Translational Brain Science, Department of Metabolism and Systems Science, College of Medicine and Health, University of Birmingham, Birmingham B15 2TT, UK; Department of Neurology, Queen Elizabeth Hospital, University Hospitals Birmingham NHS Foundation Trust, Birmingham B15 2GW, UK; National Institutes of Health and Care Research Birmingham Biomedical Research Centre, University Hospitals Birmingham, Birmingham B12 2TH, UK; Department of Biomedical Sciences, School of Infection, Inflammation and Immunology, College of Medicine and Health, University of Birmingham, Birmingham B15 2TT, UK; National Institutes of Health and Care Research Birmingham Biomedical Research Centre, University Hospitals Birmingham, Birmingham B12 2TH, UK

**Keywords:** mTBI, post-concussion syndrome, biofluids, biomarkers

## Abstract

Mild traumatic brain injury can disrupt brain function and is associated with high morbidity and healthcare utilization. While many individuals recover from mild traumatic brain injury, a significant proportion experience long-term sequelae, collectively known as post-concussion syndrome. Symptoms of post-concussion syndrome include headache, dizziness, insomnia, cognitive processing difficulties and mental health disturbances. The disease burden is augmented by the current lack of objective measures to accurately predict long-term symptoms and deficits, providing an opportunity to utilize biomarkers in biofluids. A large proportion of available diagnostic clinical tools are subjective symptom scores. This review aims to explore current fluid biomarkers, grouped by clinical symptoms. With the available literature, we have discovered a wide range of fluid biomarkers that have been investigated for predicting post-traumatic headache, including neuropeptides; sleep disturbances, such as cortisol and melatonin; vestibular disturbances, including interleukin-6 and neurone-specific enolase; and vomiting, such as S100B. Along with physical symptoms, biomarkers investigated for predicting cognitive disturbances include inflammatory markers, S100B, neurofilament light chain, tau, microRNA and hormones. Biomarkers to predict mental health disturbances may include brain-derived neurotrophic factor, tau and cortisol. By utilizing such biomarkers, there is capacity to adopt a personalized medicine approach to facilitate early interventions for those most in need while also identifying individuals with a favourable prognosis who can safely return to their normal activities.

## Teaser

Lyons *et al*. review current evidence on biofluid biomarkers that may predict persistent symptoms following mild traumatic brain injury. They highlight specific markers linked to physical, cognitive and mental health disturbances, suggesting that these could support more personalized care and early intervention strategies in affected individuals.

## Graphical Abstract

**Graphical Abstract:**
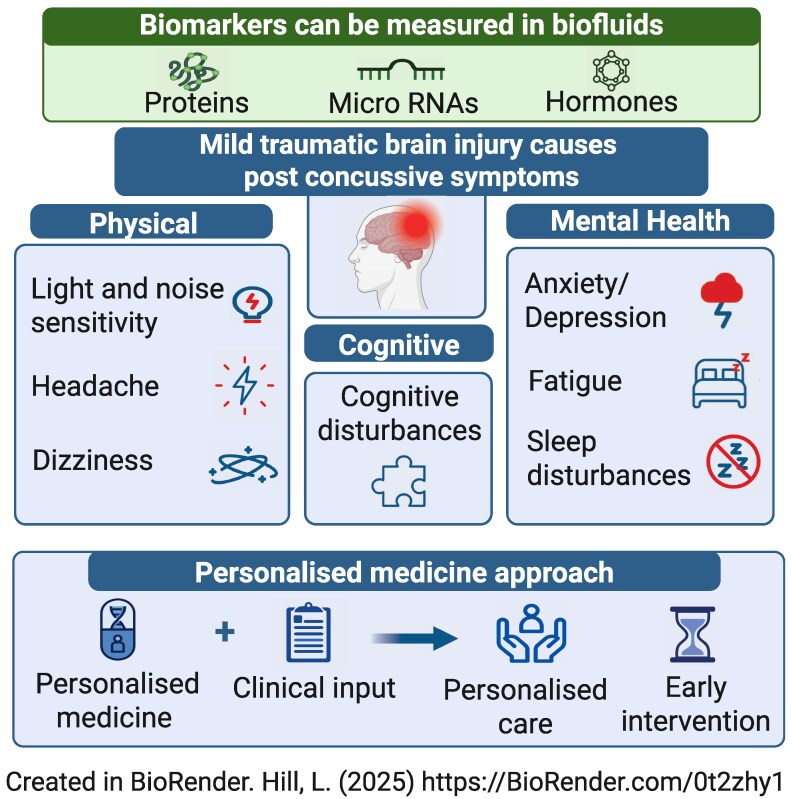

## Introduction

**Figure 1:**
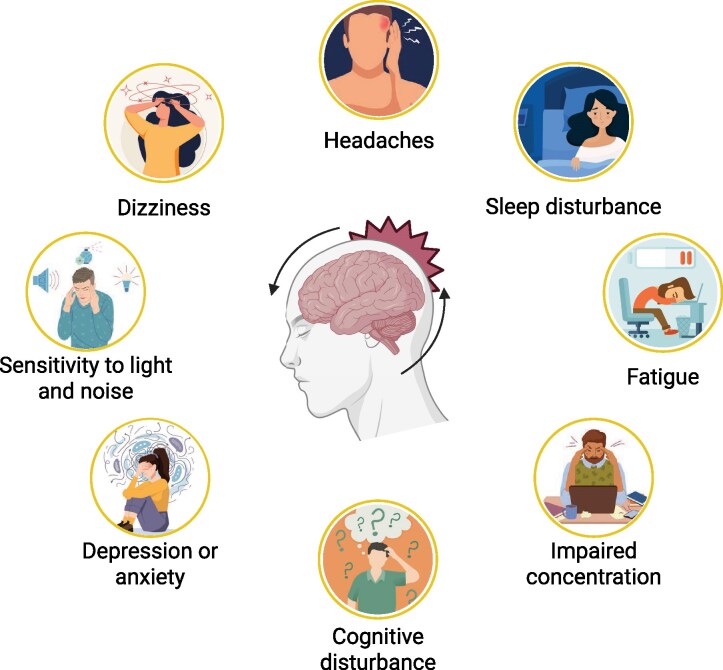
**An infographic detailing the symptoms of PCS.** These include headaches, sleep disturbances, fatigue, impaired concentration, dizziness, sensitivity to light and noise, depression or anxiety and cognitive disturbances. Created in BioRender. Hill, L. (2025) https://BioRender.com/wdqs6nn.

Traumatic brain injury (TBI) refers to an alteration in brain function or other evidence of brain pathology caused by an external force, which results in tissue damage.^1^ It is typically classified into mild, moderate and severe categories. Mild TBI (mTBI) is commonly referred to as a concussion or minor head injury, which can temporarily disrupt brain function and is increasingly associated with significant morbidity.^2^ While many individuals recover from mTBI, a significant proportion experience long-term sequelae. These symptoms are collectively known as post-concussion syndrome (PCS) (Fig. 1).^3^ According to the International Classification of Diseases 10th edition (ICD-10) definition, PCS consists of three out of eight symptoms and functional changes, including headache, dizziness, insomnia, irritability, fatigue, difficulty concentrating, memory difficulties and intolerance of stress, emotion and/or alcohol.^4^ The National Institutes of Health (NIH) declared mTBI as a major public health problem in 1999.^5^

Persistent PCS refers to the persistence of symptoms beyond 3 months and is estimated to affect 15% of patients with mTBI, although due to the lack of objective prognostic measures for PCS, this figure may underestimate the prevalence, particularly regarding cognitive disturbances. Persistent PCS has lasting effects on executive function, cognition, memory and learning.^6^ Advanced neuroimaging studies reveal cortical structure involvement, particularly the frontal–temporal area, post-mTBI.^7^ Additionally, repetitive concussions have been associated with neurodegenerative diseases, such as chronic traumatic encephalopathy, Alzheimer's disease and amyotrophic lateral sclerosis, emphasizing the long-term consequences of mTBI.^8-11^ Predictors of poorer outcomes at 12 months include a history of brain injury, medical comorbidities, living alone, being non-white, female and usage of alcohol or certain medications.^12^ Consequently, due to its high prevalence, mTBI leads to substantial morbidity and increased healthcare utilization.^13^

The diagnosis of mTBI is primarily confirmed by a medical professional. This assessment often employs the Department of Veterans Affairs and Department of Defense (VA/DoD) diagnostic criteria, but can also be diagnosed using the ICD-10, American Congress of Rehabilitation Medicine (ACRM) or Mayo criteria (Table 1).^14^ Currently, there are no objective measures, such as biomarkers, available to accurately quantify the extent of brain injury following mTBI. Furthermore, the ability to accurately predict long-term complications remains unestablished; the primary factor consistently predicting delayed recovery from mTBI is the severity of acute and subacute symptoms.^15^ Therefore, there is an urgent need to develop accurate and reproducible objective biomarkers for mTBI that can predict long-term consequences. These biomarkers should be applicable for immediate assessment at the time of injury and over subsequent months and would be useful to supplement clinical judgement. Using biomarkers as an independent diagnosis refers to the capacity of biomarkers to determine the severity of TBI and predict the probability of long-term health risks based on objective physiology, rather than solely relying on subjective patient-reported symptoms or clinical judgement. In the future, the independent use of biomarkers may augment the clinician's decision-making to distinguish mTBI from overlapping symptom profiles, such as post-traumatic stress disorder or chronic pain.

**Table 1:** Different systems for classifying mTBI

	VA/DoD	ICD-10	ACRM	MAYO
Loss of consciousness	0–30 min	0–30 min	0–30 min	0–30 min
Alteration of consciousness/mental state	<24 h	A moment up to <24 h	Immediately following injury	
Post-traumatic amnesia	<24 h	<24 h	<24 h	<24 h
Glasgow Coma Scale (best available within 24 h)	13–15		13–15	13–15
Neuroimaging	Normal	Normal	Normal or abnormal^a^	Depressed, basilar or linear skull fracture (dura intact)
Other			Acute neurologic sign(s) Physical, cognitive, emotional symptoms <72 h	If none of the criteria for moderate–severe TBI are met Only need one of more of the criteria for LOC, PTA or neuroimaging changes

VA/DoD, Veterans Affairs Department of Defence; ICD-10, International Classification of Diseases 10th Revision; ACRM, American Congress of Rehabilitation Medicine; TBI, traumatic brain injury; LOC, loss of consciousness; PTA, post-traumatic amnesia.

^a^If neuroimaging is abnormal, use mTBI ‘with neuroimaging evidence of structural intracranial injury’.

In England and Wales, head injuries account for ∼1.4 million hospital visits annually, with around 200 000 resulting in admissions.^16^ Among these hospital visits, 85% (1.2 million) are identified as mild, while 10% are classified as moderate and 5% as severe.^16^ Population-based estimates suggest that the true rate of mTBI exceeds 600 cases per 100 000 individuals.^17^ Among deployed UK military personnel, the estimated prevalence of mTBI is 4.4%, rising to 9.5% for those in combat roles.^18^ In comparison, the US military reports estimates ranging from 12 to 23%.^19,20^ Blast injuries, specifically, are the most frequent mechanism of injury, with injuries occurring from the primary blast wave along with secondary impact from debris, with blasts being recognized as a significant component of the wars in Iraq and Afghanistan.^18^ In addition, there has been significant concern in mTBI within the sports community, with suggestions that 21–38% of all TBIs occur during sporting activities and that these populations are at higher risk for multiple TBIs.^21-24^

Sport-related concussion (SRC) is now acknowledged as a significant health concern, with the potential for long-term neuropsychological impact from repetitive concussions and non-concussive incidents, particularly prevalent among athletes engaged in high-impact sports, such as rugby, American football and boxing.^25^ Timely identification and intervention following an SRC can significantly lower the chances of recurring head injuries and prolonged symptoms. Presently, sideline assessments rely on recognizing the injury, assessing symptoms and employing various neuropsychological tests, such as the Sport Concussion Assessment Tool—sixth edition (SCAT6).^26^ SRCs remain a critical issue as clinical signs and symptoms may swiftly shift or present with a delay. This complexity makes it challenging for healthcare providers, ranging from coaches and trained first aid personnel to team doctors or physiotherapists, to promptly recognize and evaluate on-field concussions; many instances go unnoticed and consequently untreated. The importance of detecting the concussive event is essential. Technologies that accurately quantify head impacts, for example through accelerometery, helmet sensors or video-based analytics, are critical for providing objective measures of the impact of the injury and may detect unnoticed concussive events. Without these objective markers, interpretations are *post hoc* and mostly speculative. These technologies alongside biomarkers will be revolutionary for the mTBI diagnostic field.

Fluid biomarkers can provide valuable insights into the underlying pathophysiological process of mTBI, along with predicting clinical outcomes and long-term sequelae. Biomarkers can be detected in various biofluids, including blood, cerebrospinal fluid (CSF), saliva, urine, hair and faeces. Most studies use blood and saliva as biomarkers, due to their non-invasive nature of collection and established protocols for processing and validation in commercially available kits and protocols. Several other biological fluids including urine, hair and stool also offer similarly applicable platforms for biomarker investigation that could better reflect systemic and neuroendocrine responses. These biofluids could be especially useful in community-based settings for longitudinal studies. They have the potential to identify individuals who are at a higher risk of developing post-concussion symptoms and could provide appropriate follow-up care and support individual management plans. By identifying such biomarkers, a personalized treatment approach can be adopted to facilitate early interventions while also identifying individuals with a favourable prognosis who may safely return to their normal activities.

**Figure 2:**
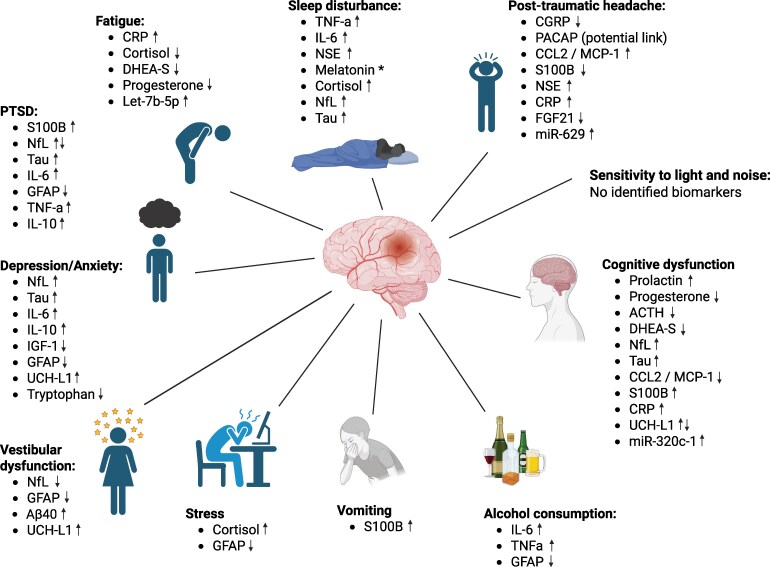
Post-concussion symptoms and associated biomarkers in blood, saliva or CSF samples. *Different to physiological patterns. ↑ statistically significant positive association with this symptom; ↓ statistically significant negative association with this symptom. ACTH, adrenocorticotropic hormone; Aβ, amyloid beta; CCL2, chemokine ligand 2; CGRP, calcitonin gene-related peptide; CRP, c-reactive protein; DHEA-S, dehydroepiandrosterone sulphate; FGF21, fibroblast growth factor 21; GFAP, glial fibrillary acidic protein; IL, interleukin; IGF-1, insulin C-like growth factor 1; MCP-1, macrophage chemotactic protein-1; MiRNA, micro-ribonucleic acid; NF, neurofilament light; NSE, neurone-specific enolase; PACAP, pituitary adenylate cyclase activating peptide; PTSD, post-traumatic stress disorder; TNFα, tumour necrosis factor-alpha; UCH-L, ubiquitin c-terminal hydrolase L1. Created in BioRender. Hill, L. (2025) https://BioRender.com/5wml85u.

This review aims to explore current mTBI-associated biomarkers in relation to their corresponding clinical outcomes. We focus on mTBI in military personnel, sports-related and paediatric injuries. Clinical symptoms are divided into physical or neurobehavioral symptoms, and biomarkers associated with these are summarized. A summary of the post-concussion symptoms and biomarkers are found in Supplementary Table 1 and in Fig. 2.

## Search strategy and selection criteria

This review adopted systematic search strategies to inform the capture of relevant literature (see Supplementary Table 2). The references for this review were identified through searches of PubMed between 1969 and 1 November 2024, and references from relevant articles. The search terms utilized are detailed in Supplementary Table 1. There were no language restrictions, and the ‘human’ filter was applied. The final selection of references was based on their relevance to the subjects discussed in this review. Studies containing a paediatric population were included if there were adults in the cohort also.

## Physical symptoms

There are various physical symptoms that can occur after a mTBI, including headache, sleep disturbances, vestibular disturbances and vomiting. Multiple studies investigate biomarkers and show correlations to clinical symptoms and predictors of outcomes following a mTBI.

## Post-traumatic headache

Post-traumatic headache (PTH) is a frequent outcome of mTBI, although the reported incidence of PTH attributed to mTBI varies, ranging from 16 to 79% at 3 months and 15 to 58% at 1 year.^27-30^ The International Classification of Headache Disorders, third edition (ICHD-3), characterizes PTH as a new or worsening headache resulting from head injury and occurring within 7 days of trauma, following the recovery of consciousness or the ability to sense and report pain.^31^ PTH may manifest independently or as part of PCS.^32^ It can be categorized as acute, if the headache resolves within 3 months, or persistent, if it continues beyond 3 months.^31^ PTH most commonly presents with a migraine-like phenotype, typically with sensitivity to light and sound, along with a throbbing one-sided headache.^33^ However, imaging studies demonstrate structural differences between migraine and PTH, including regional volumes, surface area, brain curvature and cortical thickness.^34,35^

### Neuropeptides

Calcitonin gene-related peptide (CGRP) is a neuropeptide that plays a crucial role in both migraine and PTH. It has been implicated in the initiation and persistence of migraine headaches, with elevated CGRP levels measured during the attack phase in both serum^36,37^ and saliva.^38,39^ Provocation studies involving CGRP infusion have also demonstrated the ability to induce migraine-like attacks in individuals prone to migraines.^40,41^

Although there is a definite link between CGRP and PTH, limited research exists on the mechanism of action of CGRP in PTH; some suggest that it shares similar mechanisms with migraine.^42^ Intravenous (IV) infusion of CGRP has been demonstrated to worsen headaches in individuals with persistent PTH, displaying migraine-like features.^42,43^ Treatment with CGRP monoclonal antibodies has also been shown to be effective in PTH, with up to 28% of patients experiencing at least a 50% reduction in their monthly headache days (moderate to severe) after using this medication.^44^ Conversely, a study by Ashina *et al.*^45^ found lower CGRP plasma levels in participants with persistent PTH attributed to mTBI (i.e. mTBI at least 12 months before study participation) compared to healthy controls. Another longitudinal study found no correlation between CGRP levels and either PTH post-mTBI or headache burden, following military deployment.^46^ Notably, Eggertsen *et al.*^47^ discovered elevated serum CGRP (median 3.9 months after mTBI) in individuals with persistent PCS. Intriguingly, higher CGRP levels were associated with fewer headache days, shorter headache duration and reduced current headache pain at baseline. It is worth noting that CGRP is a challenging neuropeptide to analyse, due to its very short-half-life (<10 min in blood) and inconsistent sample collection protocols, which may attribute to differences in findings across studies.

There is a hypothesis that links reduced plasma levels of CGRP in PTH patients and the persistence of daily headaches.^47^ This hypothesis posits that continuous headache experiences may lead to a depletion of CGRP in trigeminal afferents. However, it is important to note that the studies discussed in this review primarily focused on persistent PTH rather than the acute phase.^45,47^ Thus, further research is warranted to elucidate the precise role of CGRP in the context of concussion during the acute setting.

Pituitary adenylate cyclase activating peptide (PACAP) is another neuropeptide present in trigeminal afferents alongside CGRP.^48,49^ PACAP exists in two bioactive forms, PACAP-38 (90% constitute in mammalian tissue) and PACAP-27, and serves as a hormone, neurotransmitter and neuromodulator.^50^ There have not been any studies measuring PACAP in the context of PTH, following an mTBI. However, a Danish provocation study demonstrated hypersensitivity to PACAP-38 in persistent PTH attributed to mTBI.^51^ While not in the context of mTBI populations, conflicting findings have emerged regarding PACAP in individuals prone to migraines, as some studies indicate lower levels compared to healthy controls.^52^ Tuka *et al.*^52^ discovered a significantly lower concentration of PACAP-38 in patients with migraine during the interictal period (phase between migraine attacks) compared to healthy controls, with concentrations increasing during migraine attacks. Furthermore, the infusion of PACAP has been shown to induce migraine-like headaches.^53-55^ The exploration of PACAP in mTBI is justified due to its parallel role with CGRP in migraines, potential involvement in persistent PTH and the limited yet promising research indicating its ability to provoke PTH.

### Other biomarkers

S100B is a Ca^2+^ binding protein highly expressed by astrocytes and is being extensively studied as a potential biomarker in mTBI.^56,57^ A Dutch study demonstrated that patients with headache had a significantly lower median S100B concentration in their serum (within 6 h of mTBI) than those without headache at first examination post-trauma.^58^ However, in the same cohort study, they demonstrated that elevated levels of acute serum S100B and neurone-specific enolase (NSE) were associated with headaches 6 months after presentation. This association was significant for NSE but not for S100B.^59^

Interestingly, in a prospective cohort study of 52 children with concussion (aged 7–21 years with a mean age of 14 years), salivary miR-629 (collected within 14 days of head injury) was positively associated with ‘I have headaches’ 4 weeks after injury.^60^ However, concussion was assessed using the Sport Concussion Assessment Tool (SCAT3) symptom score based on parent or self-report 4 weeks after injury, which is not a conclusive test. It is not clear whether these are headaches related to the head injury *per se*. Moreover, in chronic migraine patients, changes in miR-629 in plasma samples were different depending on treatment response.^61^

Niu *et al.*^62^ found that elevated serum levels of chemokine ligand 2 (CCL2), also known as macrophage chemotactic protein-1 (MCP-1), assessed at 7 days and 3 months post-injury, independently posed a risk for greater impact of headaches in mTBI patients. In SRC, it was reported that headaches showed a significant negative correlation with the expression of serum fibroblast growth factor 21 (FGF21), within 3 months of injury.^63^

High-sensitivity C-reactive protein (hsCRP) is commonly used to detect and monitor inflammation. Individuals with mTBI who have elevated serum hsCRP (>1.10 mg/L, measured within 30 days post-injury) have reported a significantly higher incidence of headaches at their initial presentation.^64^ However, a separate study observed no correlation between elevated baseline plasma hsCRP levels in acute mTBI and the persistence of headaches up to 3 months (one headache per week).^65^ These conflicting results suggest that it remains inconclusive as to whether hsCRP is specific to PTH, and these inconsistent results may be due to inadequate controls for biological and methodological variability.

## Sleep disturbances

Sleep disturbances, including daytime sleepiness, difficulties falling or staying asleep and reduced quality, are common following mTBI.^66-69^ Crucially, these issues can persist long term, with 50% of patients reporting sleep difficulties at 3 months and 33% at 12 months,^68^ and some experiencing issues up to 3 years post-injury.^70^ Insomnia is one of the most common sleep disorders after mTBI and is referred to as difficulty falling asleep, staying asleep or waking up too early.^71^ Sleep disturbances may develop soon after injury, due to a primary insult to the sleep–wake pattern and/or further down the line, during the subacute and chronic stages.^21^ This primary insult can damage certain regions of importance in the brain, such as the hypothalamus, brainstem, retinohypothalamic tract (pathway involved in circadian rhythm, transmitting light levels to the hypothalamus) and reticular activating system (network of nerve pathways that plays a role in the sleep–wake transition).^72^ Secondary damage may include structural, genetic and biochemical alterations to the sleep–wake mechanism.^71^ Both melatonin and cortisol are released in a diurnal pattern and are involved in the body's circadian rhythm (sleep–wake cycle).^73^ In addition, fatigue is a significant and often overlooked symptom post-mTBI. It can have a profound effect on quality of life and contribute to overall cognitive performance.^74^

### Melatonin

Melatonin is a naturally occurring hormone that is commonly used as a marker of the circadian cycle^73^ and has been identified as a potential marker of mTBI. For example, Maerlender *et al.*^75^ looked at youths with concussion and found that a lower bedtime urinary melatonin concentration correlated with increased sleep awakenings (number and duration) and reduced sleep efficiency. Furthermore, there were no differences between urine melatonin concentration at bedtime and at 30 min post-waking in individuals with mTBI compared to the control group, although there was a significant increase upon waking compared to controls.^75^ Barlow *et al.*^76^ did not find any difference in overnight urinary melatonin production in children with mTBI relative to healthy controls. However, Ayalon *et al.*^77^ found that in individuals with chronic insomnia post-mTBI, the peak salivary melatonin was delayed by greater than 2 h compared to the normal circadian cycle in healthy controls. Collectively, these studies suggest that urinary melatonin may provide valuable insights into the underlying pathophysiology of sleep-related disturbances and help guide melatonin-modulating interventions for patients with mTBI.

### Cortisol

Cortisol, also known as the ‘stress hormone’, typically displays very low levels in blood and urine in the evening followed by a sharp rise in the early morning and a peak at typical awakening. Additionally, there is a transient increase 30–60 min after typical waking [cortisol awakening response (CAR)], followed by a steady decline throughout the day.^78,79^ Studies have suggested that mTBI can trigger alterations in cortisol levels and its diurnal rhythm, which can influence sleep disturbances. In response to the acute phase post-mTBI, the hypothalamic–pituitary–adrenal (HPA) axis activates and leads to an increase in cortisol levels.^80^ Prolonged cortisol elevation can be detrimental to cognitive function, mental health and sleep, potentially affecting the overall recovery trajectory of an individual.^79,80^

Interestingly, Daneva *et al.*^81^ found that salivary cortisol levels were significantly elevated post-mTBI from 4 h to 4 days post-injury when compared to healthy controls. Subjective sleep questionnaires were also administered at baseline and Day 4, which showed more sleepiness and insomnia symptoms, with significant improvement at Day 4. However, this study did not look at cortisol levels or sleep disturbances beyond 4 days post-injury. Villegas *et al.*^78^ found similar bedtime and waking cortisol levels compared to controls, but individuals with mTBI had a blunted CAR at baseline (average 6–7 days post-injury) and at a further 1-week follow-up, which they identified as a significant predictor of symptom severity. Conversely, some studies have found no significant differences in cortisol levels when compared to healthy controls.^75,82^ Cortisol shows substantial promise as a useful biomarker in mTBI, due to increasing evidence for its link to altered diurnal rhythm following injury, and the associated detrimental effects of this prolonged cortisol elevation on sleep.

### Other biomarkers

In addition to the most prominent sleep-related hormones mentioned above, there are other biomarkers that have been suggested to correlate with sleep disturbances following an mTBI. Pro-inflammatory cytokines in the blood, such as interleukin-6 (IL-6) and tumour necrosis factor-alpha (TNFα), have been reported to be elevated in mTBI. A study involving post-9/11 veterans described a considerable prevalence of blast exposure (78%, 430/550 participants) and reported incidents of at least one mTBI in 69% of the participants. Upon analysis of fasted plasma samples, it was found that IL-6, TNFα and NSE had significant positive correlations with Pittsburgh Sleep Quality Index (PSQI) global scores, in which a higher score indicates poorer sleep.^83^ Interestingly, the average time since their most recent mTBI was 9.15 years, suggesting long-term changes in proteins following TBI and potentially specifically blast exposure. In US military investigations, NfL and, to a lesser extent, tau were linked to chronic PCS symptoms and poor sleep quality.^84^ The Chronic Effects of Neurotrauma Consortium (CENC) study, which focused on war fighters with mTBI, observed a significant elevation of plasma NfL and a trend towards elevated plasma tau in the mTBI cohort with poor sleep quality.^85^

Inflammatory proteins may also act as biomarkers for mTBI and sleep disturbances. Shetty *et al.*^64^ reported that patients with mTBI who showed high levels of serum hsCRP (>1.10 mg/L) experienced more fatigue symptoms. In patients with sports-related concussion, fatigue symptoms were negatively associated with urine cortisol levels, along with dehydroepiandrosterone sulphate (DHEA-S) and progesterone.^86^ Finally, salivary miRNA could be a potential biomarker for sleep disturbances in concussion, with particular promise due to its non-invasive collection. In children with concussion, higher levels of salivary let-7b-5p were associated with a response to ‘I get tired a lot’ at 4 weeks post-head injury.^60^

There is growing evidence that mTBI affects the visual system, including retinal nerve fibre layer (RNFL) thinning,^87^ yet few studies have explored biomarkers that could predict this. There were no publications that used biomarkers to predict visual outcomes after mTBI; however, this would be an exciting avenue for studies to explore in the future.

These findings provide valuable insights into the relationship between biofluid biomarkers, effects on sleep and clinical outcomes in paediatric, sport and military populations with mTBI.

## Vestibular disturbances

mTBI stands as a prominent cause of chronic disability in young adults. In particular, post-injury vestibular dysfunction serves as a crucial indicator of reduced return-to-work rates, with a reduction from 75 to 33% at 6 months post-injury.^88,89^ Multiple causes contribute to chronic dizziness after concussion, encompassing central (e.g. infarcts or haemorrhage) and peripheral vestibular disorders (e.g. benign paroxysmal positional vertigo, vestibular neuronitis, and labyrinthine concussion).^89^ The manifestation of vertigo and imbalance is often intricate, involving a combination of central and peripheral factors.

In addition, following exposure to overpressure events, specifically blast injuries, active-duty army personnel with self-reported dizziness displayed reduced levels of glial fibrillary acidic protein (GFAP) and NfL in acute blood samples.^90^ Intriguingly, the levels of GFAP and NfL were reduced after a primary blast injury, whereas the usual trend in individuals exposed to direct impact mTBI is increased GFAP and NfL.^90^ Elevated ubiquitin c-terminal hydrolase (UCH-L1) and Aβ40 were also observed in those with increased dizziness.^90^ Additionally, a Dutch study suggested there may be a positive association between increased NSE in blood taken within 6 h of injury and dizziness severity, though not statistically significant.^59^

## Vomiting

A study of 104 patients with mTBI showed no significant correlation between serum biomarkers and symptoms such as nausea or dizziness. However, patients who vomited exhibited higher S100B levels (0.50 µg/L) than those who did not (0.25 µg/L), suggesting a potential association between post-traumatic vomiting and increased S100B, which warrants further investigation.^58^ S100B could rise in serum due to transient tissue hypoperfusion and cellular stress, which can cause astrocyte injury and release S100B from the brain into the bloodstream. Vomiting is unlikely to cause direct brain injury, but severe or prolonged vomiting can lead to systemic stress and potential fluid loss, resulting in secondary S100B elevation.

## Neuro-behavioural symptoms

In addition to physical symptoms, mTBI can affect neuro-behavioural symptoms that include cognitive disturbances, post-traumatic stress disorder, depression and anxiety and stress and alcohol misuse. They are typically assessed using clinical questionnaires and scoring systems, as outlined in Table 2.

**Table 2:** Summary of clinical questionnaires and tests

Questionnaire	Abbreviation	Clinical symptom	Summary
PTSD checklist military version	PCL-M	Stress/post-traumatic stress disorder	To assess the presence and severity of post-traumatic stress disorder symptoms in military personnel
Neurobehavioral Symptom Inventory	NSI	PCS, dizziness, nausea, vision, hearing, headaches, fatigue, depression, anxiety, cognitive disturbance	A 22-item self-report questionnaire of common PCS symptoms to track symptoms over time
Patient Health Questionnaire-9	PHQ-9	Depression	A self-administered questionnaire for the diagnosis of common mental disorders, including depression
Center for Epidemiological Studies Depression	CES-D scale	Depression	A 20-item self-reporting depression scale that asks about restless sleep, feeling lonely and poor appetite
Depression Anxiety Stress Scales	DASS	Depression, anxiety, stress	A 42-item self-reporting questionnaire
De Utrechtse Coping List Questionnaire	UCL	Coping	Questionnaire to evaluate coping strategies
The Rivermead Post-Concussion Symptoms Questionnaire	RPQ	PCS: headaches, dizziness, nausea, sleep disturbances, fatigue, depressed, poor memory, vision	Questionnaire to assess PCS
Pittsburgh Sleep Quality Index	PSQI	Sleep	A self-reporting questionnaire that assesses sleep quality over a 1-month interval
D-KEFS Color-Word Interference Test	CWIT	Cognitive disturbance	A neuropsychological test of processing speed and executive function
Traumatic Brain Injury Quality of Life	TBI-QOL	Mobility, headache, fatigue, mental health, cognitive disturbances and social functioning	Measures physical, emotional, cognitive and social aspects of health-related quality of life

D-KEFS, Delis–Kaplan Executive Function System; PCS, post-concussion syndrome.

## Cognitive disturbances

mTBI often results in cognitive deficits, affecting working memory, verbal learning, attention, processing speed and executive functions.^91,92^ While many recover within 3 months, up to 58% experience lasting impairments contributing to PCS.^6^ If not effectively managed, PCS can persist for months or years, impacting quality of life and contributing to secondary decline in mental health.^93^ Neurocognitive tests can help identify cognitive impairment, but these face limitations due to biases from unreliable baselines and inadequate prognostic efficacy.^6,94^ Here, we explore the emerging field of fluid biomarkers and their ability to be utilized as prognostic tools for cognitive outcomes in mTBI.

### Inflammatory markers

In SRC, low serum FGF21 is linked to impaired impulse control.^63^ MCP-1 is also implicated, with reduced levels correlating with a higher number and severity of cognitive symptoms, including feelings of fogginess, poor balance and delayed reaction time.^63^ Elevated serum IL-4, copeptin, cathepsin D, receptor for advanced glycation end products (RAGEs) and neuropilin-1 are associated with impulsivity-related traits in mTBI.^95^ Conversely, serum interferon alpha-2 (IFNα2) and soluble CD40L are negatively associated with impulsivity.^95^ However, this study did not find significant differences in NfL levels between mTBI patients and healthy controls in the context of impulsivity.

In a prospective study, participants with acute mTBI were assessed for inflammatory cytokines within 24 h of injury, and again at 6 months post-injury, alongside neuropsychological evaluations.^96^ At 6 months, elevated plasma IL-17A levels were significantly linked to decreased executive function, measured by inhibition scores on the Delis–Kaplan Executive Function System (DKEFS) Color Word Interference Test.^96^ Another study linked increased baseline hsCRP in acute mTBI to persistent cognitive impairment at three months, particularly affecting attention and delayed recall.^65^ The CENC study, involving 195 veterans and service members post-deployment and combat exposure, found weak correlations between lower plasma TNFα levels and improved cognitive scores on the Neurobehavioral Symptom Inventory (NSI).^97^

### S100B/NSE/UCH-L1/NfL/tau

When examining S100B as a marker for cognitive impairment, Boussard *et al*.^98^ discovered that patients with neuropsychological deficits exhibited prolonged and higher S100B serum concentrations. Significant differences were observed at 2 weeks and 6 months post-mTBI compared to those without deficits. Another study linked acute serum S100B to deficits in working memory, verbal learning and fluency at 3 months, though most patients also had CT abnormalities, potentially confounding results.^99^ UCH-L1 was also investigated, showing positive correlations with verbal learning and working memory, but negative correlations with visual memory. As part of the US Defense and Veterans Brain Injury Center/Traumatic Brain Injury Center of Excellence (TBICoE) 15-Year Longitudinal TBI study, 37 US service members and veterans were prospectively enrolled with mTBI. At baseline (mean 7.5 months post-injury), higher serum UCH-L1 was significantly associated with more cognitive concerns at follow-up (mean 36.2 months post-injury) utilizing the TBI quality of life questionnaire, TBI-QOL.^100^ When analysing acute S100B, patients subjected to hourly serum S100B measurements within 12 h of injury displayed a trend towards impaired neuropsychological functioning in measures of information processing speed, memory and attention.^101^ Conversely, another study found that at 6 hours post-injury, patients with or without elevated serum levels of protein S100B (>0.22) exhibited similar cognitive speed and memory function when tested 7–21 days post-injury. Both groups performed worse than healthy controls, suggesting that an elevated serum S100B level may not reliably indicate neuropsychological recovery in mTBI.^102^

In a study of boxers, 23 of 30 boxers had elevated CSF NfL within 6 days of a head injury, and following 2 weeks of rest, 12 of 26 still showed elevated NfL and slower processing speed (four lost to follow-up).^103^ This is supported by an Australian cohort study that measured blood samples in football players with SRC compared to uninjured and musculoskeletal controls, from 24 h to 26 weeks post-injury. While no significant associations were found between GFAP or NfL levels and cognitive outcomes overall, in the SRC group, moderate GFAP levels at 26 weeks showed a borderline significant link to slower identification task (latency), while extreme and minimally elevated NfL levels at 26 weeks were significantly associated with slower identification task (latency), but moderate NfL levels were not.^104^ The lack of consistent associations with moderate NfL levels suggests that this relationship may vary. In active boxers, longitudinal measurements of NfL and tau were not associated with cognitive performance, though concussive events were not well tracked.^105^ However, a military study investigating veterans with repetitive mTBI found significant positive correlations between exosomal tau and NSI cognitive symptoms, as well as plasma tau and symptom scores related to mood and overall symptom burden.^106^ Conversely, a prospective study involving active service military showed no significant relationship between plasma tau or Aβ42 and neurocognitive performance in mTBI.^107^ Finally, in a study of symptomatic former NfL players at high risk of chronic traumatic encephalopathy, lower levels of CSF Aβ1–42 were associated with poorer episodic memory performance.^108^

### Micro-ribonucleic acid

Micro-ribonucleic acids (MiRNAs) are small, noncoding molecules that play a regulatory role in protein synthesis and can indicate disease pathology. In a prospective study of male collegiate football athletes, pre-season analysis revealed a negative correlation between neurocognitive scores and serum miRNAs (miR-20a, miR-505, miR-195 and miR-151-5p).^109^ When comparing pre- and post-season miRNA concentrations, players starting with higher neurocognitive scores exhibited significant increases in miR-505, miR-486, miR-30d, miR-92a, miR-362-3p and miR-195. The concentration of miRNA-92a significantly increased from pre- to post-season. However, with only two (10%) players diagnosed with concussion, caution is needed in drawing definitive conclusions.^109^ Similarly, a paediatric study found that salivary miR-320c-1 was positively associated with ‘I have problems remembering what people tell me’, suggesting a link to concentration or memory deficits.^60^

### Hormones

In a study involving 95 athletes, including 26 with SRC, cognitive symptoms were significantly associated with various hormone concentrations, within 7 days of injury.^86^ Cognitive symptoms included feeling slowed down, ‘in a fog’, ‘don't feel right’, difficulty concentrating, difficulty remembering and confusion. Symptoms negatively correlated with peripheral blood levels of adrenocorticotropic hormone (ACTH), cortisol, DHEA-S and progesterone and positively correlated with prolactin. These findings suggest a potential link between cognitive symptoms and neuroendocrine dysfunction. Notably, no differences were observed between athletes with concussions and healthy athletes, suggesting that these variations may not be attributed to concussion but rather to different phenotypic profiles.^86^

## Post-traumatic stress disorder

The decline in mental health following a concussion is now widely acknowledged as a significant issue, impacting daily life and overall well-being for months after their injury.^110^ Recent studies report that around 20% of individuals experience post-traumatic stress disorder or major depressive disorder (MDD) within 6 months of a mTBI, a notably higher rate compared to those with non-head orthopaedic injuries.^111^ These investigations also identify prior mental health conditions and injury circumstances as key predictors of post-concussion mental health outcomes.^111,112^

Concussion-related psychiatric disorders are now recognized as distinct subtypes of traditional mental health conditions. These conditions may not respond as effectively to standard antidepressant medications and psychotherapy when compared to non-concussion–related disorders.^113,114^ A retrospective cross-sectional study that used advanced brain mapping techniques has identified TBI-associated depression as separate from primary major depressive disorder, revealing unique connectivity patterns and underlying mechanisms associated with depression after TBI.^115^ This underscores the idea of distinct subtypes of depression and post-traumatic stress disorder and the need for tailored approaches to better understand and address these conditions in the context of mTBI.

### Tau

In a study involving 42 US military personnel who reported mTBI within 18 months, significantly elevated levels of exosomal tau and Aβ42 were observed in neuronal-derived exosomes from the peripheral blood, compared to healthy controls.^116^ However, while the association between post-traumatic stress disorder and exosomal tau levels showed a trend, it did not reach statistical significance.^116^ Further research into exosomal p-tau-181 in 195 veterans with repeated mTBI found a weak, significant correlation with the total PCL-M score (PTSD checklist military version). Additionally, exosomal tau levels were significantly associated with both the total PCL-M score and its affective sub-domain on the NSI.^106^ Plasma tau levels also showed a significant correlation with the PCL-M score, suggesting the potential utility of tau as a biomarker for post-traumatic stress disorder severity in repeated mTBI cases.^106^ In a study that involved 102 service members and veterans, those with both chronic mTBI (average time since injury: 46 months) and post-traumatic stress disorder exhibited significantly elevated tau levels in blood plasma compared to those with mTBI alone and injured controls without post-traumatic stress disorder.^117^ Sleep medication use and post-traumatic stress disorder severity were independent predictors of tau concentrations, with higher levels observed in non-users of sleep medications and those with moderate to severe post-traumatic stress disorder symptoms.^117^

### Interleukins

In a US military study involving personnel who reported mTBI within 18 months, elevated IL-10 levels in neuronal-derived exosomes were associated with greater post-traumatic stress disorder severity.^116^ Similarly, a prospective study of individuals with acute mTBI found that elevated IL-10 at 6 months post-injury were significantly linked to more severe post-traumatic stress disorder symptoms measured by the PCL scale.^96^ In a cohort of post-9/11 veterans, where 58.73% were diagnosed with post-traumatic stress disorder, plasma IL-6 positively correlated with post-traumatic stress disorder symptom severity.^83^ Together, these findings suggest that blood serum IL-10 and IL-6 levels may have utility as indicators that relate to the severity of post-traumatic stress disorder symptoms in individuals with recent mTBI. Further work is needed to better define the interplay between pro- and anti-inflammatory cytokines and their potential as clinical biomarkers across in different post-traumatic stress disorder populations.

### GFAP

In the study with post-9/11 veterans, plasma GFAP showed a negative association with post-traumatic stress disorder severity.^83^ The TRACK-TBI study, which evaluated adults with mild to moderate TBI (GCS 13–15, 36.4% with positive CT scans), found that participants with probable post-traumatic stress disorder (PCL-5 ≥ 33) at 6 months had significantly lower plasma GFAP levels on the day of injury.^118^ This suggests a potential protective role of GFAP against the development of post-traumatic stress disorder in this population.

### NfL/cortisol/S100B

The CENC study included 195 veterans and service members with lifetime mTBI screening following deployment and combat exposure.^97^ Post-traumatic stress disorder symptom severity, as measured by PCL-M scores, was moderately positively correlated with exosomal NfL, weakly with plasma TNFα and plasma NfL and marginally with exosomal IL-6.^97^ In contrast, a separate military study reported a negative correlation between serum NfL and PCL-hyperarousal.^119^

In a cohort of 88 patients with mTBI, serum cortisol levels measured at admission and 7 h post-injury did not show a significant correlation with post-traumatic stress disorder symptoms.^120^ However, a noteworthy finding was the significant link between serum S100B concentrations at 10 h post-injury and post-traumatic stress disorder symptoms at the 1-year follow-up.^120^

## Depression/anxiety

Similar biomarkers, associated with depression and anxiety, are summarized below.

### Tau

A recent study compared CSF biomarkers between 68 symptomatic former NfL players at high risk of chronic traumatic encephalopathy and 21 controls.^108^ On average, the former NfL players were 21.8 years removed from their last concussion. Findings revealed that higher CSF p-tau181 levels correlated with more severe depression among players. However, no significant differences emerged between players and controls with respect to t-tau, p-tau181, Aβ1–42 or soluble triggering receptor expressed on myeloid cells 2 (sTREM2) biomarkers.^108^ A study by Gill *et al.*^116^ found no relationship between depression and tau levels from neuronal-derived exosomes from peripheral blood in military personnel with an mTBI. Another investigation, involving 109 military personnel and veterans, predominantly with mTBI (94.1%), revealed that serum tau was positively associated with total NSI and PHQ-9, as well as the PCL-Negative Mood subscales.^119^ Furthermore, a study focusing on veterans with repeated mTBI showed that plasma tau was significantly associated with the total PHQ-9, as well as correlating with total NSI.^106^ Within the TBICoE study, baseline serum tau (mean 7.5 months post-injury) was significantly associated with anxiety at follow-up (mean 36.2 months post-injury using the TBI-QOL anxiety scale).^100^

### Interleukins/GFAP

A study focusing on deployed military personnel suggested a tendency for depression to be linked with higher IL-10, an anti-inflammatory cytokine, in neuronal-derived exosomes from the peripheral blood.^116^ Vedantam *et al.*^96^ also found that higher plasma IL-10 at 6 months post-injury was significantly associated with more pronounced depression symptoms (CES-D) in acute mTBI. Among the post-9/11 veterans, 27.27% were diagnosed with mood disorders and 18.73% had anxiety disorders. Plasma levels of IL-6, a predominantly inflammatory cytokine, showed a positive correlation with both depressive symptoms and NSI scores.^83^ Moreover, plasma IL-10 demonstrated a positive correlation with depression symptoms, whereas GFAP had a negative correlation with anxiety (DSM-IV).^83^

### NfL/BDNF/IGF-1/UCH-L1

In the CENC study involving veterans, correlations were observed between the PHQ-9 and both exosomal and plasma NfL.^97^ In a separate investigation of 331 patients with mTBI, serum insulin-like growth factor-1 (IGF-1) within the first week displayed a significant negative correlation with depression and anxiety scores at both the initial and sixth week post-injury. Additionally, lower serum IGF-1 observed at the sixth week correlated with higher depression and anxiety scores.^121^ A study of 42 college athletes, half of whom were cleared to play after mTBI while the other half served as controls, did not establish a significant correlation between brain-derived neurotrophic factor (BDNF) in serum and saliva and their scores on DASS.^122^ The TBICoE study found at follow-up (mean 36.2 months post-injury) that baseline serum UCH-L1 (mean 7.5 months post-injury) was significantly associated with depression (TBI-QOL). There were clinically meaningful associations between higher levels of baseline serum UCH-L1 and a deterioration of anxiety and depression from baseline.^100^

### Kynurenines

A sub-study of the AIM-TBI study recruited 338 patients with mTBI and collected acute bloods in ED. They looked at the relationship of three kynurenines (tryptophan, xanthurenic acid, and picolinic acid) with clinical outcomes. There was a significant relationship found between lower acute plasma tryptophan and higher depression scores (HADS-D) at 6 months post-injury, but not anxiety scores (HADS-A).^123^

## Stress/alcohol use

### GFAP and TNFα

In a study focused on post-9/11 veterans, a negative association was demonstrated between plasma levels of GFAP and stress levels (measured by DASS), alongside alcohol use.^83^ Conversely, plasma TNFα and IL-6 were positively correlated with lifetime drinking. The study did not find significant associations for plasma levels of AB40/AB42, BDNF, tau, NfL or pNF-H10 with the various mental health and alcohol use outcomes measured in this population.^83^

### Cortisol

Spikman *et al.*^124^ investigated the interplay of coping styles and chronic cortisol levels in 46 patients diagnosed with mTBI. Their study revealed that patients with higher levels of passive coping, measured by the Dutch Utrechtse Coping List (UCL-Pas), both at 2 weeks and 6 months post-injury, exhibited lower chronic cortisol levels in hair samples.^124^ Similarly, those employing more avoidant coping (UCL-Avoi) displayed lower chronic cortisol levels pre-injury at 2 weeks, plus both pre- and post-injury at 6 months. These findings persisted even after the exclusion of patients with pre-injury depression, indicating a notable association between coping styles and cortisol regulation in individuals with mTBI.^124^ In a separate study, 26 university athletes with concussions were followed over 2 years, with researchers finding no significant difference in cortisol levels between concussed and non-concussed athletes.^82^ However, there was a notable correlation between acute cortisol levels and perceived stress among the concussed athletes.^82^

## Limitations and future directions

There are a few limitations of the literature and this review article that need to be addressed. We have also posed future recommendations for studies looking at biomarkers in biofluids and PCS.

### Limitations of the literature

The current literature reveals several notable gaps in our understanding of mTBI and its associated outcomes. There is a lack of studies that have investigated the potential of biofluids as predictive biomarkers for visual outcomes following mTBI, especially since retinal nerve fibre layer (RNFL) thinning, which is associated with vision loss, can occur post-mTBI.^87^ Moreover, studies often report general measures, such as the total score of symptom questionnaires including the NSI or RPQ, without further extrapolating their subdomains. This limitation hinders our ability to explore potential associations between specific features of PCS. We recommend that future studies should correlate biomarkers with the total symptom burden and specific domains, such as vestibular or cognitive domains, among others.

Furthermore, to date, most studies have focused on analysing potential biomarkers in blood and more recently saliva; however, there is minimal research focused on the use of urine, hair or faeces as potential fluids for assessing mTBI and its associated outcomes. These biofluids offer unique opportunities for non-invasive biomarker analysis, yet their potential remains largely unexplored in the context of mTBI. Further investigations in this area could provide valuable insights into the development of novel biomarker approaches for mTBI diagnosis, prognosis and treatment monitoring.

Presently, research on mTBI fluid biomarkers primarily focuses on male cohorts, likely due to the recruitment of military personnel in these studies. There is minimal consideration given to the influence of hormonal factors or the menstrual cycle on biomarker profiles. Earlier studies have shown substantial fluctuations in biomarkers across various phases of the menstrual cycle.^125^ This underscores the necessity for future investigations to incorporate the effects of the menstrual cycle on biomarker profiles, ensuring that emerging tests are relevant for both male and female populations. We recommend future studies investigating biomarkers and mTBI account for both genders due to these important implications that can affect biomarker concentrations and induce variability.

Understanding the association between mTBI and specific PCS is crucial for exploring the distinct challenges that arise following such injuries, including headaches, sleep disturbances, dizziness, cognitive impairments and mental health issues. These symptoms may result from changes in the CNS and brain tissue function following mTBI or may be influenced by the injury context itself and its associated effects, such as dizziness or cognitive deficits. However, establishing a clear cause-and-effect relationship based on the timeline of events is challenging due to the broad and unpredictable nature of mTBI outcomes and associated polytrauma. It is difficult to establish in a clinical setting as psychological and somatic symptoms often stem from overlapping sources, such as concussion, psychiatric stressors and systemic injury, among others, which can all alter the biomarker profile. The occurrence of polytrauma in concussed patients can introduce diverse injury contexts, potentially complicating the accurate correlation between isolated mTBI and the subsequent development of these symptoms, particularly those related to mental health.

### Limitations of this review

While a systematic search strategy was employed, several limitations inherent to a narrative review design should be acknowledged. A narrative review does not include formal quantitative synthesis or meta-analyses, which limits the ability to draw definite conclusions about effect sizes or causal relationships. As only one search engine was used to identify literature, it is possible that some relevant studies were not captured. Heterogeneity across study designs, outcome measures and participant populations also limits the ability to compare findings. This review primarily focused on biomarkers demonstrating significant associations with a broad range of symptoms, and the time points at which these biomarkers were collected were discussed within the main text. However, future reviews focusing specifically on biomarker timing and related outcomes would provide valuable insight into their temporal relationships. A formal risk-of-bias assessment was not conducted, as it falls outside the scope of a narrative review. Consequently, the conclusions presented here should be interpreted as a synthesis of existing evidence intended to highlight emerging patterns and guide future research, rather than conclusive or generalizable findings.

### Future directions

In recent years, the advancement of biomarkers stands out as a leading focus in concussion research, particularly in the military and sporting populations. The ability to accurately predict concussion with biomarkers and prevent individuals returning to play or duty who are likely to have long-term side effects is invaluable. However, many of these biomarkers currently lack the capability to independently diagnose SRCs. There are multiple non-invasive pitch side tests for concussion emerging, including a recent FDA-approved Abbots portable iSTAT® TBI cartridge that assesses levels of UCH-L1 and GFAP in whole blood of patients who have had a concussion and produces results within 15 min.^126^ These rapid, pitch-side biomarker tests will be particularly useful for high-intensity gameplay, as distinguishing between a safe tackle and a significant impact proves exceedingly challenging with current subjective concussion tests.

Alongside the exploration of fluid biomarkers, it becomes imperative to develop technologies capable of identifying potential concussive incidents with high accuracy.^127^ The development of such technologies would furnish a more comprehensive understanding of how the intensity of a head impact influences both biomarker responses and symptom manifestation. Ultimately, this advancement could pinpoint athletes who warrant additional evaluation, thereby enhancing medical protocols and treatment approaches.

## Conclusion

mTBI is a complex condition with diverse clinical presentations, often leading to a myriad of symptoms that can have significant negative impacts an individual's quality of life. In this narrative review, we have explored the potential of fluid biomarkers in predicting the outcomes of mTBI, organized according to the various clinical symptoms and manifestations experienced by patients.

The collective findings from this review emphasize the significant potential for biofluid biomarkers in predicting and elucidating the complex relationship between mTBI and the onset and severity of PCS. Fluid biomarkers offer promising avenues for enhancing diagnostic precision, prognostic capabilities and the development of personalized treatment strategies for individuals at risk of PCS following an mTBI.

## Supplementary Material

fcaf501_Supplementary_Data

## Data Availability

Data sharing is not applicable as no primary data were generated from this review.

## Appendix

UK mTBI-Predict Consortium

Adam Hampshire, Agata Czarnecka, Ahmed Fouad Abdel-Hay, Aimee R. Smith, Alex Bryant, Alexandra J. Sinclair, Ali Mazaheri, Alice J. Sitch, Aliyah Mannan, Altus Chan, Andreas Yiangou, Andrew P. Bagshaw, Andrew Palmer, Angus M. Hunter, Animesh Ghose, Asha Strom, Caroline W. Mugo, Carl R. Krynicki, Caroline Witton, Cherie Nicholls, Chloe N. Thomas, Claire H. Brown, Clare Anderson, Dan Ford, Danny Smullen, David J. Smith, David Jimenez-Grande, Davinia Fernandez-Espejo, Eleanor G. Rowan-MacIndoe, Emma C. Lardner, Hamid Dehghani, Hannah Fisher, Hannah S. Lyons, Hyojin Park, Ian Varley, Jacob H. Tennant, James L. Mitchell, Jan Novak, Jennie Gavin, Jessica C. Hubbard, John T. Read, Jonthan J. Deeks, Julita Sulkowska, Karen J. Mullinger, Karen Tester, Katherine L. Cox, Katie Morris, Linda Martina Coughlan, Lisa J. Hill, Maria Balaet, Mark Thaller, Matt Hill, Mia Mann, Nasreen Akhtar, Ned J. Jenkinson, Neil Winkles, Pete J. Hellyer, Raymond F. Reynolds, Richard J. Blanch, Ryan S. Ottridge, Sabrina Qureshi, Samuel J.E. Lucas, Sarah Berhane, Syama Mohan, Thomas Meredith, Tom Inns and Yousef F. Hyder
