# Supplementary material for: Biofluid-based predictors of post-concussion symptoms: a narrative review of mild traumatic brain injury biomarkers
Source: Brain Commun. 2025 Dec 18;8(1):fcaf501. doi: 10.1093/braincomms/fcaf501 (PMC12820430; doi:10.1093/braincomms/fcaf501)
Supplement: fcaf501_Supplementary_Data [file fcaf501_supplementary_data.pdf]

Supplementary Table 1: Comprehensive Overview of Biomarkers Investigated in Mild Traumatic Brain Injury. Mild traumatic brain injury, mTBI; post-traumatic headache, PTH; calcitonin gene-related peptide, CGRP; pituitary adenylate cyclase activating peptide, PACAP; chemokine ligand 2, CCL2; macrophage chemotactic protein-1, MCP-1; S100B; neuron-specific enolase, NSE; c-reactive protein, CRP; neurofilament light, NfL; interleukin-6, IL-6; tumour necrosis factor-alpha, TNFα; glial fibrillary acidic protein, GFAP; amyloid beta, Aβ; ubiquitin c-terminal hydrolase L1, UCH-L1; fibroblast growth factor 21, FGF21; interleukin-4, IL-4; CD40L, CD40 ligand; receptor for advanced glycation end products, RAGE; interferon alpha-2, IFNα2; micro ribonucleic acid, MiRNA; adrenocorticotropic hormone, ACTH; hypothalamic-pituitary-adrenal axis, HPA; dehydroepiandrosterone-sulphate, DHEA-S; IGF-1 insulin-like growth factor 1. Key: ↑ statistically significant positive association with this symptom; ↓ statistically significant negative association with this symptom. Location: ✓ literature exists of biomarker measurements in this type of fluid; (✓) shouldn't be found in this type of fluid typically and presence may indicate disease; ? not yet tested amongst the literature

*These studies look at persistent PTH (headache >3 months from injury), not acute PTH (defined in this review as less than 3 months from injury). # represents tryptophan metabolites

Biomarker	Structure	Significant association mTBI symptom	Production	Location						Role	Neurological Marker
				Blood	CSF	Urine	Saliva	Hair	Faeces		
**CGRP**	Neuropeptide	Persistent PTH*↓[1-3]	Trigeminal ganglion, dorsal root ganglia, PNS sensory nerve terminals, CNS	✓	✓	✓	✓	?	?	Vasodilation, pain modulation, neurotransmission, inflammation, cardiovascular regulation.	Migraine
**PACAP**	Neuropeptide	Potential link with PTH.	Mainly PNS sensory nerve terminals, CNS, endocrine system, immune cells	✓	✓	✓	✓	?	?	Neurotransmission, neuromodulation, vasodilation, inflammation, hormonal regulation, cell growth, neuroprotection.	Its role as a definitive marker not firmly established
**CCL2/ MCP-1**	Chemokine	Persistent PTH ↑[4]<br>Poor balance↓[5]<br>Cognitive disturbance↓[5]	Monocytes, macrophages, endothelial cells, epithelial cells, fibroblasts, smooth muscle cells	✓	✓	✓	✓	?	?	Recruitment of monocytes to sites of injury and inflammation.	Inflammation
**S100B**	Calcium-binding protein	Acute PTH↓[6]<br>Vomiting↑[7]<br>Cognitive disturbance↑[6,8]<br>PTSD↑[9]	Mainly astrocytes (type of glial cell in CNS)	✓	✓	✓	✓	?	✓	Intracellular calcium regulation, cellular differentiation and proliferation, neurotrophic effects, inflammation and	Glial cell injury

										immune response, glial cell function support.	
**NSE**	Neuronal cytoplasmic enzyme	Chronic PTH↑[6]<br>Sleep disturbances↑[10]	Neuronal cells	✓	✓	✓	✓	?	?	Upregulated to maintain homeostasis when axons are damaged.	Neuronal damage
**CRP**	Acute-phase protein	Acute PTH↑[11]<br>Fatigue↑[11]<br>Cognitive disturbance ↑[12]	Liver	✓	✓	✓	✓	?	?	Immune and inflammatory responses.	Inflammation
**Melatonin**	Neurohormone	Sleep disturbances↓[13]	Pineal gland	✓	✓	✓	✓	✓	?	Regulate sleep-wake cycle, free radical scavenger and antioxidant, and facilitator of core temperature and cortisol cycles.	Circadian rhythm
**Cortisol**	Steroid hormone	Sleep disturbances↑[14]<br>Cognitive disturbances↓[15]<br>Fatigue ↓[15]<br>Coping ↓[16]<br>Stress↑[17]	Adrenal glands	✓	✓	✓	✓	✓	?	Stress response, metabolism and immune system regulation, anti-inflammatory, blood pressure regulation, circadian rhythm	Stress response, circadian rhythm
**NfL**	Intermediate filament (cytoskeletal protein)	Sleep disturbances↑[18,19]<br>Dizziness↓[20]<br>Cognitive disturbances ↑[21-23]<br>PTSD↑[24] ↓[25]<br>Depression↑[24]	Cell body and axons of neurons	✓	✓	✓	✓	?	?	Axonal structural support	Axonal injury

**Tau**	Microtubule-associated protein	Sleep disturbances ↑[18,19]<br>Cognitive disturbances↑[26]<br>PTSD↑[26,27]<br>Depression↑[25,26,28]<br>Anxiety↑[23]	Axons	✓	✓	✓	✓	?	?	Stabilises microtubules in axons	Axonal injury
**IL-6**	Pro-inflammatory cytokine	PTSD↑[10]<br>Depression↑[10]<br>Sleep disturbances↑[10]<br>Alcohol consumption↑[10]	Various cells including immune cells.	✓	✓	✓	✓	?	✓	Induction of acute phase response, regulation of immune response, and haematopoiesis, tissue repair and regeneration.	Inflammation and dysregulation of immune system
**IL-10**	Anti-inflammatory cytokine	PTSD↑[29,30]<br>Depression↑[10,30]	Various cells including immune cells.	✓	✓	✓	✓	?	✓	Anti-inflammatory, immunoregulation, tissue repair	Inflammation and dysregulation of immune system
**TNFα**	Pro-inflammatory cytokine	Sleep disturbances↑[10]<br>PTSD↑[24]<br>Alcohol consumption↑[10]	Various cells including immune cells.	✓	✓	✓	✓	?	✓	Primarily regulation of immune responses, cell proliferation, apoptosis, induction of inflammation.	Inflammation, infection, and dysregulation of immune system
**GFAP**	Intermediate filament (cytoskeletal protein)	Dizziness ↓[20]<br>Cognitive disturbance↑[22]<br>PTSD↓[10,31]<br>Anxiety↓[10]<br>Stress↓[10]<br>Alcohol consumption↓[10]	Cell body of astrocytes	✓	✓	✓	✓	?	?	Astrocyte structural support	Astrocyte damage

**Aβ40**	Beta-amyloid peptide	Dizziness ↑[20]	Various cells, including neurons	✓	✓	?	✓	?	?	Amyloid plaques, neurotoxicity, synaptic dysfunction	Neurodegeneration (less pathogenic than Aβ42)
**UCH-L1**	Deubiquitinating enzyme	Dizziness ↑[20] Cognitive disturbance↑[8, 23] (Visual memory deficit ↓[8]) Depression↑[23]	Mainly neurons	✓	✓	✓	?	?	?	Protein turnover, neuronal function, neuroprotection, axonal transport	Neuronal injury, neurodegeneration
**FGF21**	Fibroblast growth factor	Acute PTH↓[5] Impulsivity↓[5]	Mainly liver, adipose tissue, pancreas (production)	✓	✓	?	?	?	?	Metabolic regulation, energy homeostasis, adipose tissue function, neuroprotective, anti-inflammatory effects	Neuroprotective
**IL-4**	Type 2 helper T cell cytokine	Impulsivity↑[32]	Mainly activated T cells	✓	✓	✓	✓	?	✓	Immunoregulator, allergic responses, tissue repair, anti-inflammatory	Neuroinflammation, immunoregulator, neuroprotective
**Copeptin**	Neuropeptide	Impulsivity↑[32]	Posterior pituitary gland	✓	✓	?	?	?	?	Osmoregulation, stress response	Surrogate marker for vasopressin release
**Soluble CD40L**	Soluble form from cleavage of membrane bound CD40L	Impulsivity↓[32]	Membrane-bound CD40L is expressed on activated T cells. sCD40L release into extracellular space.	✓	✓	?	✓	?	X	Immune activations, pro-inflammatory, platelet activation, tissue remodelling	Neuro-inflammation, neurodegenerative diseases.
**Cathepsin D**	Lysosomal aspartyl	Impulsivity↑[32]	Mainly Lysosomes	✓	✓	✓	✓	?	?	Protein degradation, tissue remodelling, hormone processing,	Neurodegeneration, inflammation

	protease enzyme									apoptosis, neuropeptide processing	
**RAGE**	Transmembrane receptor protein	Impulsivity↑[32]	Various cell types	✓	✓	✓	✓	?	?	Inflammation, induction of oxidative stress, angiogenesis, pro-apoptosis	Cellular stress, inflammation, tissue damage
**Neuropilin-1**	Transmembrane protein	Impulsivity↑[32]	Various cell types	✓	?	✓	?	?	?	Axon guidance, angiogenesis, immune modulation	Angiogenesis, axons guidance, neuroinflammation
**IFNα2**	Interferon protein	Impulsivity↓[32]	Immune cells, primarily leukocytes	✓	✓	✓	✓	?	✓	Antiviral response, immunomodulation, antiproliferative effects, induce apoptosis, modulation of cytokines	Immune response
**MiRNA**	Small, single-stranded RNA molecules	Headache↑[33] Fatigue↑[33] Cognitive disturbances↑[33,34]	Cell nucleus of various cell types	✓	✓	✓	✓	✓	✓	Regulation of gene expression	Neurodegeneration, inflammation
**ACTH**	Peptide hormone	Cognitive disturbances↓[15]	Anterior pituitary gland	✓	✓	?	?	✓	?	Stimulation of cortisol production	Adrenal function and HPA-axis
**DHEA-S**	Steroid hormone	Cognitive disturbances↓[15] Fatigue ↓[15]	Adrenal glands mainly	✓	✓	✓	✓	✓	✓	Precursor for sex hormones, adrenal function, immune system modulation, anti-inflammatory	Adrenal function, neuroprotective and anti-inflammatory effects
**Progesterone**	Steroid hormone	Cognitive disturbances↓[15] Fatigue ↓[15]	Ovaries	✓	✓	✓	✓	✓	?	Menstrual cycle, pregnancy, neuroprotective, anti-inflammatory	Neuroprotective and anti-inflammatory effects
**Prolactin**	Peptide hormone	Cognitive disturbances↑[15]	Anterior pituitary gland	✓	✓	✓	?	✓	?	Immune system modulation, lactation,	Hypothalamic and pituitary function

										regulation of reproductive function.	
**Tryptophan**	Essential amino acid	Depression ↓ [35]	Diet	✓	✓	✓	✓	✓	✓#	Essential building block for numerous biomolecules i.e., enzymes, serotonin neurotransmitters	Serotonin
**IGF-1**	Peptide hormone	Depression↓ [36]<br>Anxiety ↓ [36]	Liver	✓	✓	✓	✓	✓	?	Promote cell growth and division, regulate metabolism, mediate glucose uptake	Growth and development

Supplementary Table 2: Search Strategy

Level	Category	PubMed Search Terms
1.	Brain injury	("mild traumatic brain injury") OR (mTBI) OR (concussion) OR ("head injury")
2.	Biofluid	(fluid) OR (saliva) OR (urine) OR (sputum) OR (serum) OR (blood) OR (plasma) OR ("cerebrospinal fluid") OR (CSF) OR (faeces) OR (stool) OR (hair)
3.1	Vestibular	(vestibular) OR (balance) OR (dizziness) OR (dizzy) OR (hearing) OR (phonophobia) OR ("noise sensitivity")
3.2	Headache	(headache) OR (migraine) OR (PTH) OR ('post-traumatic headache') OR (cluster) OR ("trigeminal autonomic cephalalgia") OR (TAC)
3.3	Sleep	(insomnia) OR ('sleep disorders') OR ('circadian rhythm') OR (narcolepsy) OR (fatigue) OR (tiredness) OR (tired) OR (sleepiness)
3.4	Nausea and vomiting	((((Nausea) OR (sick)) OR (vomiting)) OR (vomit)) OR (nauseous)
3.5	Vision	((((vision) OR (visual)) OR (eye)) OR (eyes)) OR (eyesight)
3.6	Cognition	(((((((('cognitive changes') OR ('cognitive disturbances')) OR (concentration)) OR (dazed)) OR (sluggish)) OR (cognitive function)) OR ('brain fog')) OR ('memory impairment')) OR ('memory problem')
3.7	Mental health	("mental health") OR (mood) OR (depression) OR (anxiety) OR (psychiatric) OR ("psychiatric disorder") OR (irritability) OR (affective) OR ("affective instability") OR (impulsivity) OR (disorder) OR ("post-traumatic stress disorder") OR (PTSD) OR ("mental illness") OR (suicide) OR (suicidality)

Supplementary References

1. Ashina H, Al-Khazali HM, Iljazi A, et al. Low plasma levels of calcitonin gene-related peptide in persistent post-traumatic headache attributed to mild traumatic brain injury. *Cephalalgia* 2020; **40**(12): 1276-82.
2. Scher AI, McGinley JS, VanDam LR, et al. Plasma calcitonin gene–related peptide and nerve growth factor as headache and pain biomarkers in recently deployed soldiers with and without a recent concussion. *Headache: The Journal of Head and Face Pain* 2023; **63**(9): 1240-50.
3. Eggertsen PP, Palmfeldt J, Schytz HW, Hay D, Olsen RKJ, Nielsen JF. Serum calcitonin gene-related peptide in patients with persistent post-concussion symptoms, including headache: a cohort study. *Journal of Neurology* 2024.
4. Niu X, Bai L, Sun Y, et al. Mild traumatic brain injury is associated with effect of inflammation on structural changes of default mode network in those developing chronic pain. *J Headache Pain* 2020; **21**(1): 135.
5. Begum G, Reddy R, Yakoub KM, Belli A, Davies DJ, Di Pietro V. Differential Expression of Circulating Inflammatory Proteins Following Sport-Related Traumatic Brain Injury. *Int J Mol Sci* 2020; **21**(4).
6. De Kruijk JR, Leffers P, Menheere PP, Meerhoff S, Rutten J, Twijnstra A. Prediction of post-traumatic complaints after mild traumatic brain injury: early symptoms and biochemical markers. *J Neurol Neurosurg Psychiatry* 2002; **73**(6): 727-32.
7. de Kruijk JR, Leffers P, Menheere PP, Meerhoff S, Twijnstra A. S-100B and neuron-specific enolase in serum of mild traumatic brain injury patients. A comparison with health controls. *Acta Neurol Scand* 2001; **103**(3): 175-9.
8. de Boussard CN, Lundin A, Karlstedt D, Edman G, Bartfai A, Borg J. S100 and cognitive impairment after mild traumatic brain injury. *J Rehabil Med* 2005; **37**(1): 53-7.
9. Sojka P, Stålnacke BM, Björnstig U, Karlsson K. One-year follow-up of patients with mild traumatic brain injury: occurrence of post-traumatic stress-related symptoms at follow-up and serum levels of cortisol, S-100B and neuron-specific enolase in acute phase. *Brain Inj* 2006; **20**(6): 613-20.
10. Pierce ME, Hayes J, Huber BR, et al. Plasma biomarkers associated with deployment trauma and its consequences in post-9/11 era veterans: initial findings from the TRACTS longitudinal cohort. *Transl Psychiatry* 2022; **12**(1): 80.
11. Shetty T, Cogsil T, Dalal A, et al. High-Sensitivity C-Reactive Protein: Retrospective Study of Potential Blood Biomarker of Inflammation in Acute Mild Traumatic Brain Injury. *J Head Trauma Rehabil* 2019; **34**(3): E28-e36.
12. Su SH, Xu W, Li M, et al. Elevated C-reactive protein levels may be a predictor of persistent unfavourable symptoms in patients with mild traumatic brain injury: a preliminary study. *Brain Behav Immun* 2014; **38**: 111-7.
13. Maerlender A, Masterson C, Calvi JL, Caze T, Mathiasen R, Molfese D. Sleep and stress in the acute phase of concussion in youth. *Sports Med Health Sci* 2020; **2**(2): 109-14.
14. Daneva E, Makris K, Korompeli A, et al. Saliva cortisol levels and physiological parameter fluctuations in mild traumatic brain injury patients compared to controls. *Int J Neurosci* 2023; **133**(6): 612-20.
15. Di Battista AP, Rhind SG, Churchill N, Richards D, Lawrence DW, Hutchison MG. Peripheral blood neuroendocrine hormones are associated with clinical indices of sport-related concussion. *Sci Rep* 2019; **9**(1): 18605.
16. Spikman JM, van der Horn HJ, Scheenen ME, et al. Coping with stress before and after mild traumatic brain injury: a pilot hair cortisol study. *Brain Inj* 2021; **35**(8): 871-9.
17. Hutchison MG, Mainwaring L, Senthinathan A, Churchill N, Thomas S, Richards D. Psychological and Physiological Markers of Stress in Concussed Athletes Across Recovery Milestones. *J Head Trauma Rehabil* 2017; **32**(3): E38-e48.

18. Gottshall JL, Agyemang AA, O'Neil M, et al. Sleep quality: A common thread linking depression, post-traumatic stress, and post-concussive symptoms to biomarkers of neurodegeneration following traumatic brain injury. *Brain Inj* 2022; **36**(5): 633-43.
19. Werner JK, Shahim P, Pucci JU, et al. Poor sleep correlates with biomarkers of neurodegeneration in mild traumatic brain injury patients: a CENC study. *Sleep* 2021; **44**(6).
20. Boutté AM, Thangavelu B, LaValle CR, et al. Brain-related proteins as serum biomarkers of acute, subconcussive blast overpressure exposure: A cohort study of military personnel. *PLoS One* 2019; **14**(8): e0221036.
21. Neselius S, Brisby H, Marcusson J, Zetterberg H, Blennow K, Karlsson T. Neurological assessment and its relationship to CSF biomarkers in amateur boxers. *PLoS One* 2014; **9**(6): e99870.
22. O'Brien WT, Spitz G, Xie B, et al. Biomarkers of Neurobiologic Recovery in Adults With Sport-Related Concussion. *JAMA Netw Open* 2024; **7**(6): e2415983.
23. Lange RT, Gill JM, Lippa SM, et al. Elevated Serum Tau and UCHL-1 Concentrations Within 12 Months of Injury Predict Neurobehavioral Functioning 2 or More Years Following Traumatic Brain Injury: A Longitudinal Study. *J Head Trauma Rehabil* 2024; **39**(3): 196-206.
24. Guedes VA, Kenney K, Shahim P, et al. Exosomal neurofilament light: A prognostic biomarker for remote symptoms after mild traumatic brain injury? *Neurology* 2020; **94**(23): e2412-e23.
25. Pattinson CL, Shahim P, Taylor P, et al. Elevated Tau in Military Personnel Relates to Chronic Symptoms Following Traumatic Brain Injury. *J Head Trauma Rehabil* 2020; **35**(1): 66-73.
26. Kenney K, Qu BX, Lai C, et al. Higher exosomal phosphorylated tau and total tau among veterans with combat-related repetitive chronic mild traumatic brain injury. *Brain Inj* 2018; **32**(10): 1276-84.
27. Pattinson CL, Gill JM, Lippa SM, Brickell TA, French LM, Lange RT. Concurrent Mild Traumatic Brain Injury and Posttraumatic Stress Disorder Is Associated With Elevated Tau Concentrations in Peripheral Blood Plasma. *J Trauma Stress* 2019; **32**(4): 546-54.
28. Alosco ML, Tripodis Y, Fritts NG, et al. Cerebrospinal fluid tau, Aβ, and sTREM2 in Former National Football League Players: Modeling the relationship between repetitive head impacts, microglial activation, and neurodegeneration. *Alzheimers Dement* 2018; **14**(9): 1159-70.
29. Gill J, Mustapic M, Diaz-Arrastia R, et al. Higher exosomal tau, amyloid-beta 42 and IL-10 are associated with mild TBIs and chronic symptoms in military personnel. *Brain Inj* 2018; **32**(10): 1277-84.
30. Vedantam A, Brennan J, Levin HS, et al. Early versus Late Profiles of Inflammatory Cytokines after Mild Traumatic Brain Injury and Their Association with Neuropsychological Outcomes. *J Neurotrauma* 2021; **38**(1): 53-62.
31. Kulbe JR, Jain S, Nelson LD, et al. Association of day-of-injury plasma glial fibrillary acidic protein concentration and six-month posttraumatic stress disorder in patients with mild traumatic brain injury. *Neuropsychopharmacology* 2022; **47**(13): 2300-8.
32. Cardoso MGF, de Barros J, de Queiroz RAB, et al. Potential Biomarkers of impulsivity in mild traumatic brain injury: A pilot study. *Behav Brain Res* 2023; **449**: 114457.
33. Johnson JJ, Loeffert AC, Stokes J, Olympia RP, Bramley H, Hicks SD. Association of Salivary MicroRNA Changes With Prolonged Concussion Symptoms. *JAMA Pediatr* 2018; **172**(1): 65-73.
34. Papa L, Slobounov SM, Breiter HC, et al. Elevations in MicroRNA Biomarkers in Serum Are Associated with Measures of Concussion, Neurocognitive Function, and Subconcussive Trauma over a Single National Collegiate Athletic Association Division I Season in Collegiate Football Players. *J Neurotrauma* 2019; **36**(8): 1343-51.
35. Visser K, Ciubotariu D, de Koning ME, et al. Exploring the kynurenine pathway in mild traumatic brain injury: A longitudinal study. *J Neurochem* 2024; **168**(9): 2710-21.
36. Sung CW, Chen KY, Chiang YH, et al. Heart rate variability and serum level of insulin-like growth factor-1 are correlated with symptoms of emotional disorders in patients suffering a mild traumatic brain injury. *Clin Neurophysiol* 2016; **127**(2): 1629-38.
